# Supplementary material for: Prediction of base editor off-targets by deep learning
Source: Nat Commun. 2023 Sep 2;14:5358. doi: 10.1038/s41467-023-41004-3 (PMC10475126; doi:10.1038/s41467-023-41004-3)
Supplement: Supplementary file 1 — Supplementary Information [file 41467_2023_41004_MOESM1_ESM.pdf]

Prediction of base editor off-targets by deep learning

Chengdong Zhang, Yuan Yang, Tao Qi, Yuening Zhang, Linghui Hou, Jingjing Wei, Jingcheng Yang, Leming Shi, Sang-Ging Ong, Hongyan Wang, Hui Wang, Bo Yu, Yongming Wang

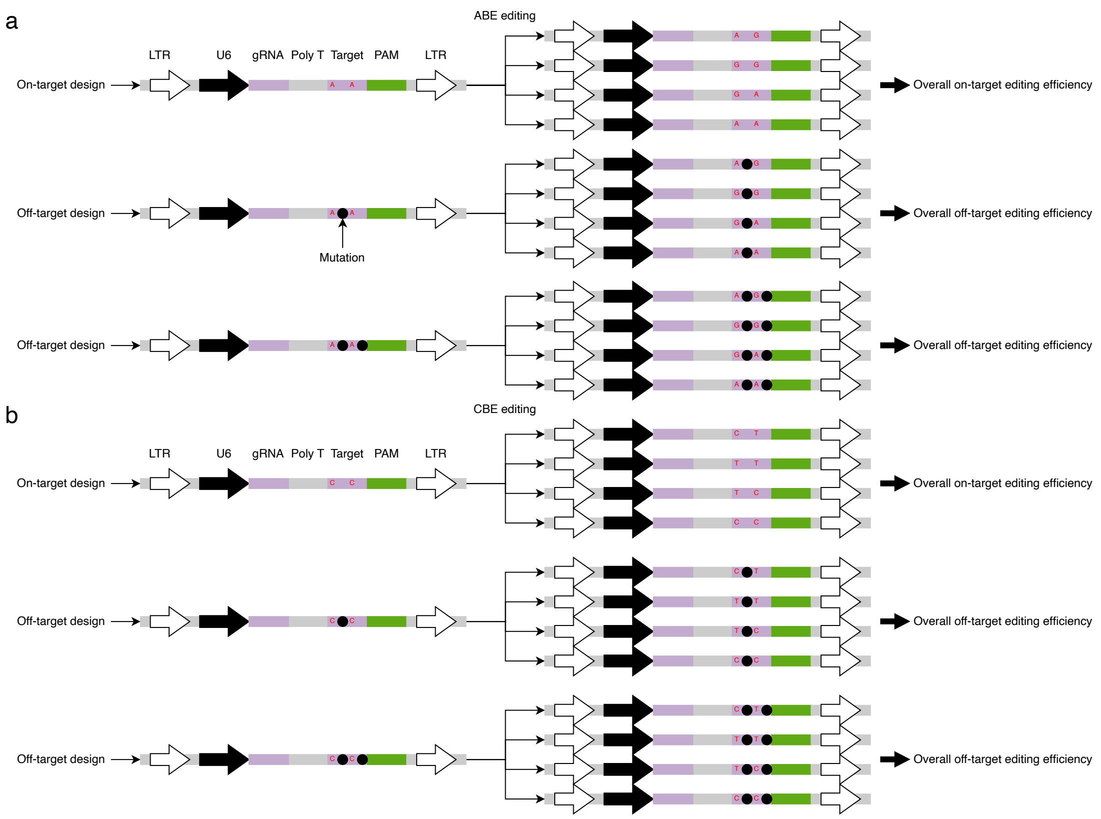

Supplementary Figure 1. The on and off-target sequence design and overall editing efficiency calculation. (a, b) Off-target sequence was designed according to on-target. After base editing (ABE or CBE), we got various specific editing results and overall editing efficiency.

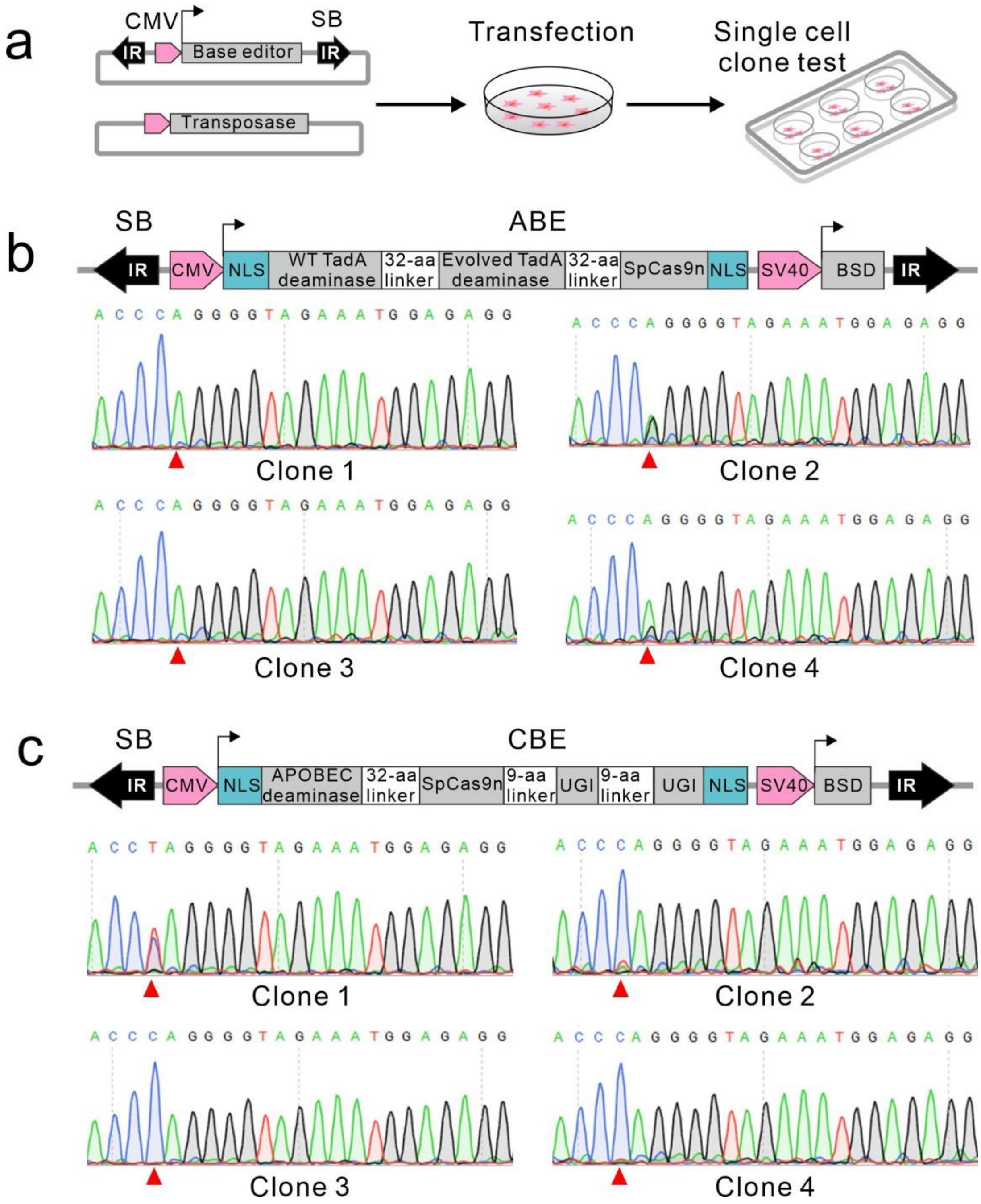

Supplementary Figure 2. Generation of single-cell-derived clones expressing base editors. (a) Schematic diagram of cell clone generation. Sleeping Beauty (SB) transposons carrying base editors together with transposase vectors were transfected into HEK293T cells, followed by blasticidin selection. Five days after transfection, single cells were isolated and seeded into a new plate for colony formation. (b, c) Test of nucleotide conversion efficiency by editing the EMX1 site in single-cell-derived clones expressing ABEmax or CBEmax. Clone #2 is selected for ABE, and Clone #1 is selected for CBE. Schematic diagrams of base editors are shown above. The edited positions are indicated by red triangles. IR: inverted repeat.

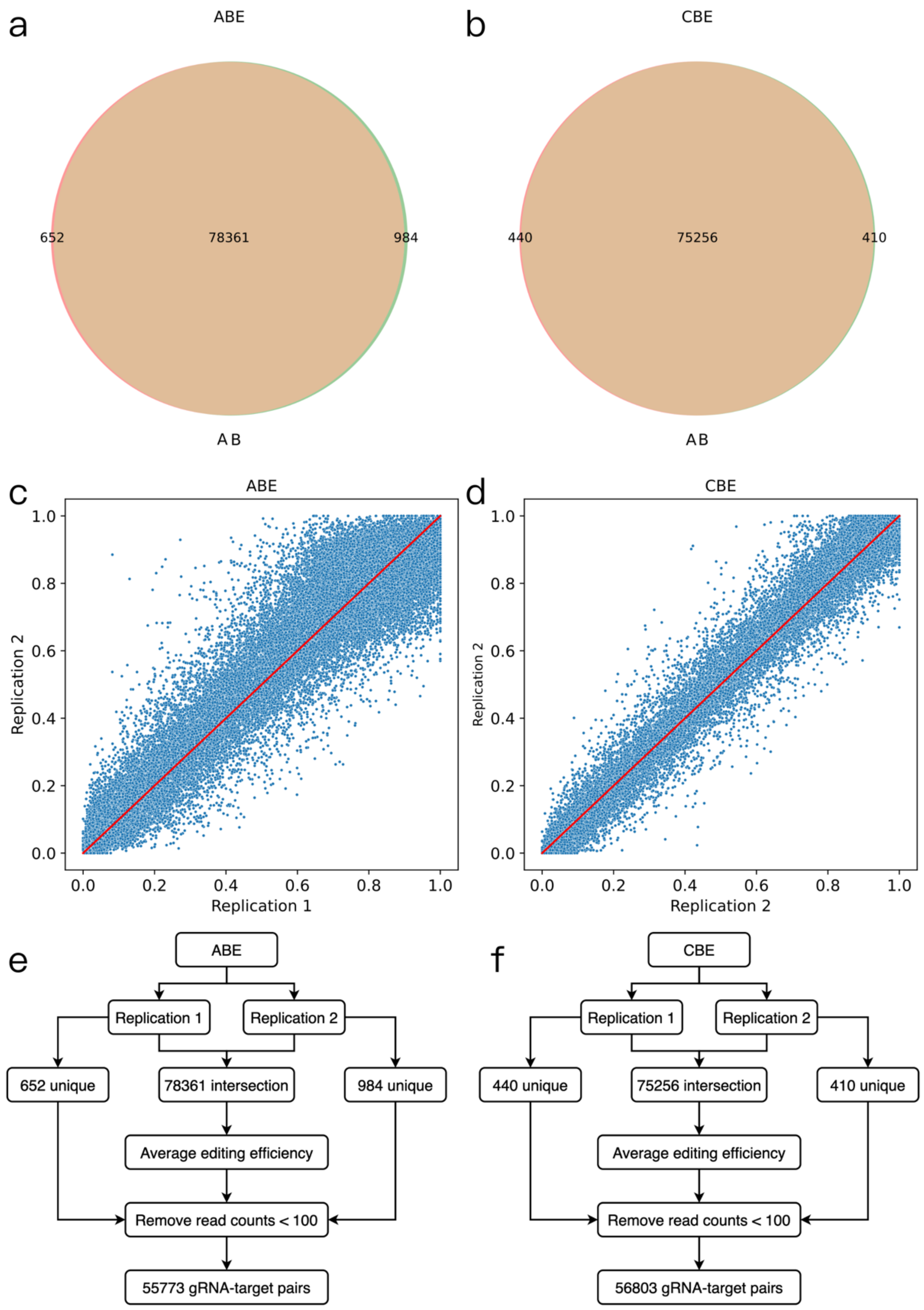

Supplementary Figure 3. The off-target screen before building a deep learning model. (a-b). Off-target relationship in two replicates, (c-d). both ABE and CBE replicates have a strong correlation. (e-f). The intersection was combined to get average editing efficiency, then remove off-target if read counts were less than 100. Source data are provided as a Source Data file.

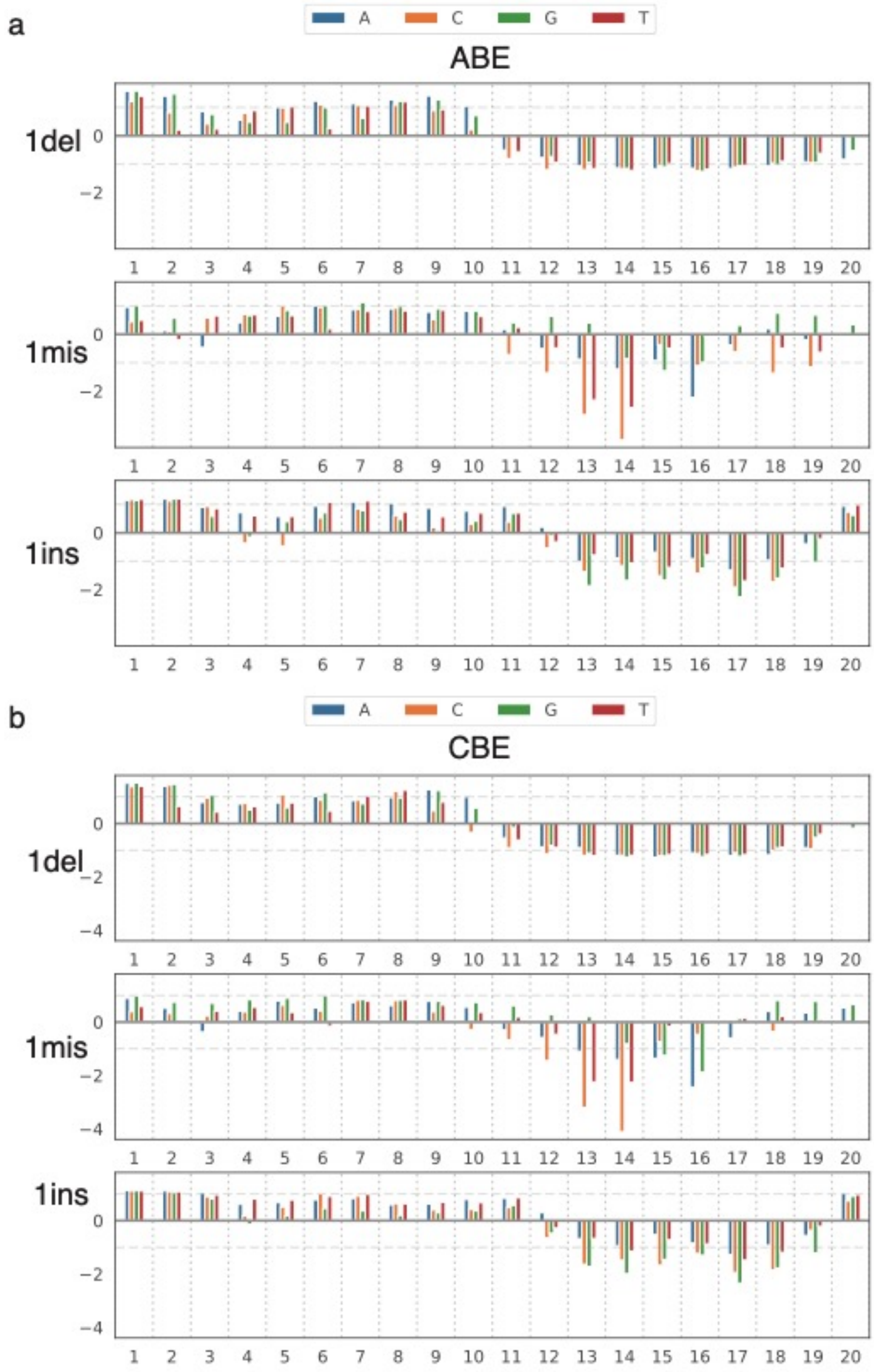

Supplementary Figure 4. The positional off-target off-on ratio for 1 bp mismatch, 1 bp insertion, and 1 bp deletion on ABE (a) and CBE (b), respectively. The position-dependent nucleotide composition of the highest 25% active gRNAs versus lowest 25% active gRNAs. Bars showed log-odds scores of nucleotide frequency for each position. The numbers below indicated the position of the nucleotides on-target DNA. Source data are provided as a Source Data file.

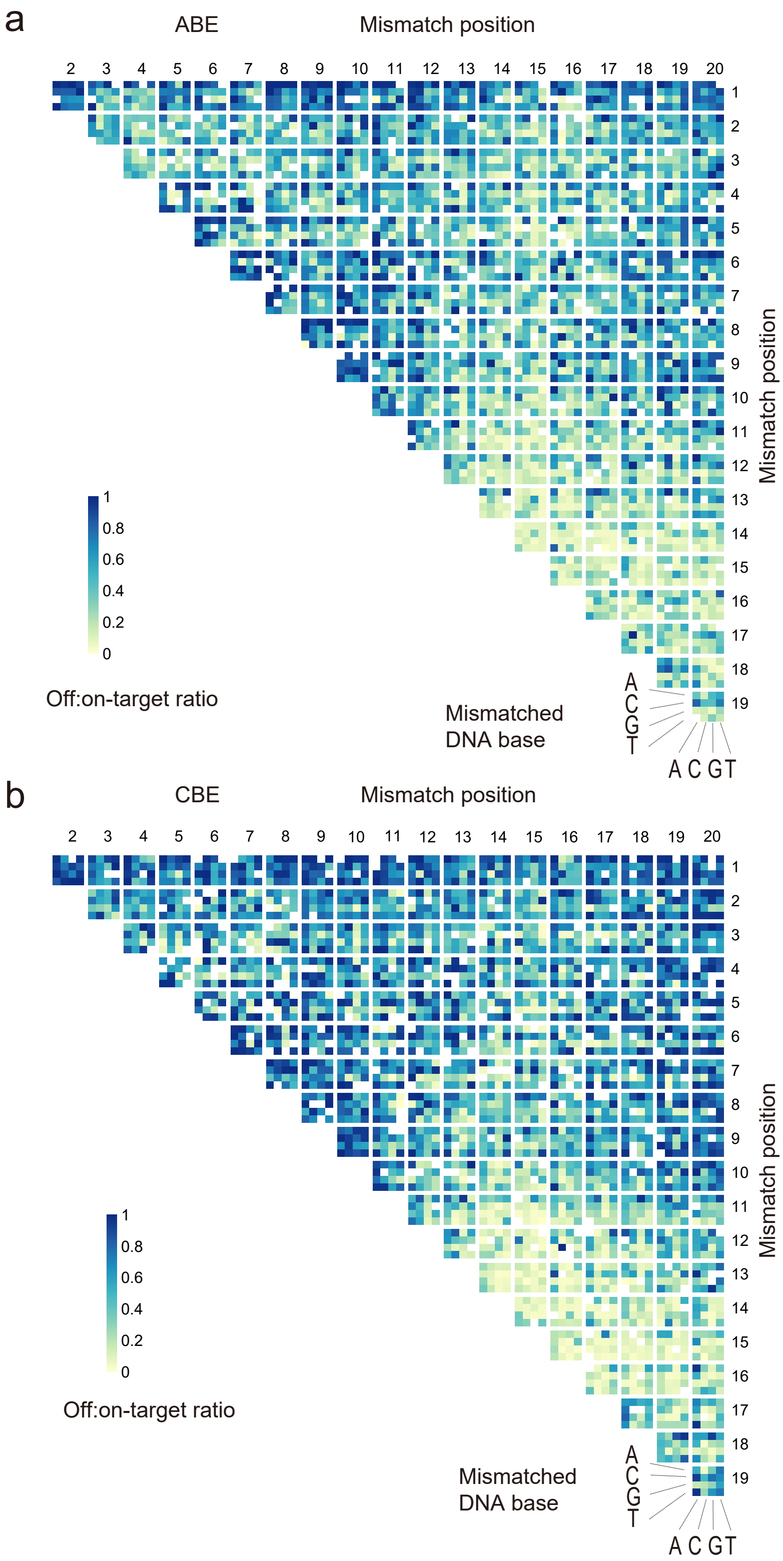

Supplementary Figure 5. Positional effects of two nucleotide mismatches on conversion efficiency. (a, b) Influence of two nucleotide mismatches on conversion efficiency for ABE and CBE, respectively. Source data are provided as a Source Data file.

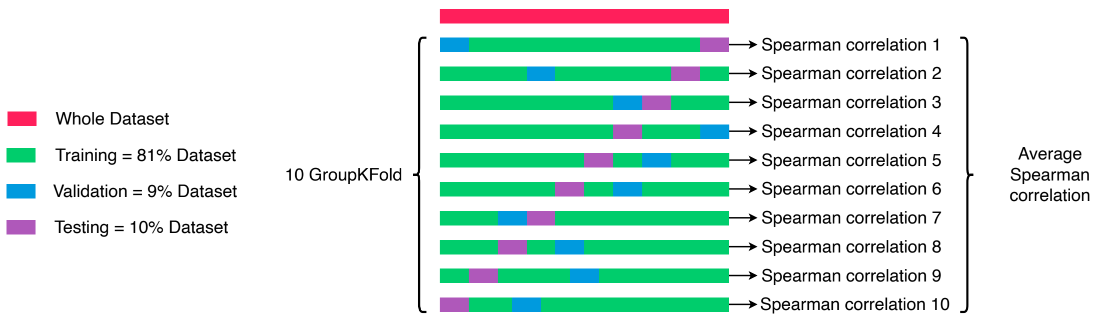

Supplementary Figure 6. 10-fold cross-validation in model training and testing. gRNA off-target pairs were grouped based on gRNA sequence.

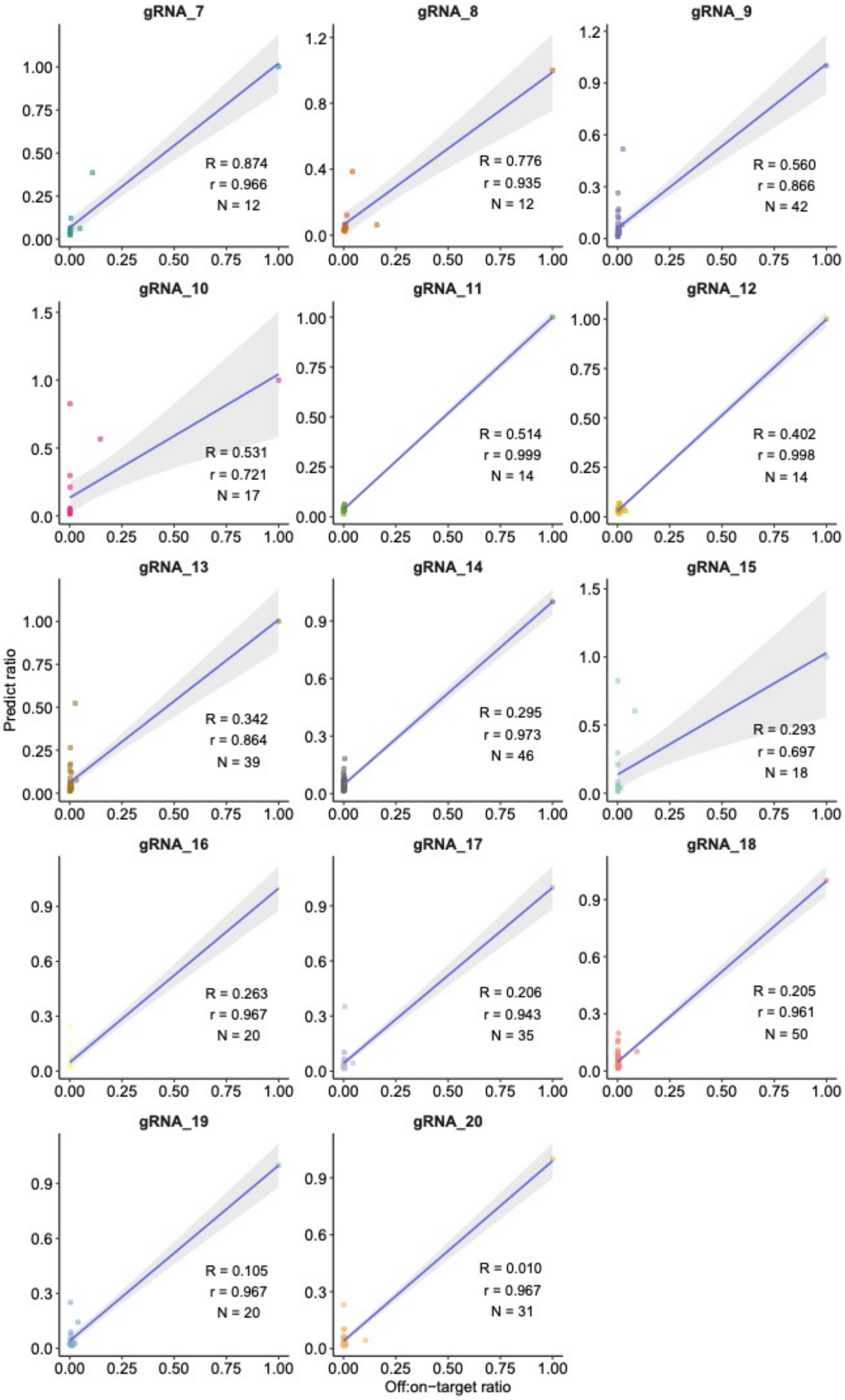

Supplementary Figure 7. Performance of ABEdeepoff for 14 groups of off-target datasets generated by Kim et al. [1]. The off and on-target efficiency ratio and predict ratio are shown. N means the number of gRNA off-target sequence pairs. Data are presented as mean predicted ratio. Source data are provided as a Source Data file.

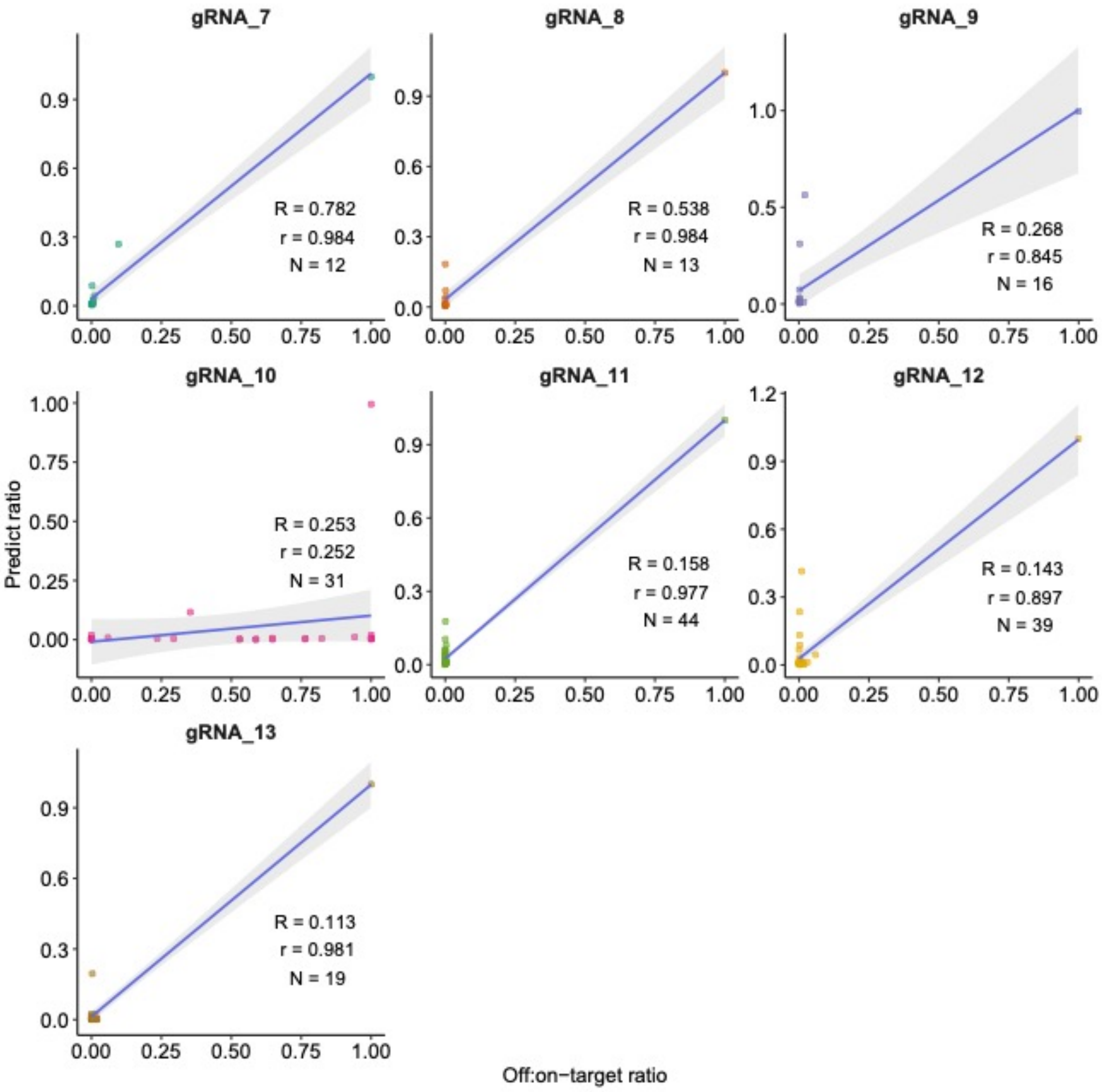

Supplementary Figure 8. Performance of CBEdeepoff for 7 groups of off-target datasets generated by Kim et al. [1]. The off and on-target efficiency ratio and predict ratio are shown. N means the number of gRNA off-target sequence pairs. Data are presented as mean predicted ratio. Source data are provided as a Source Data file.

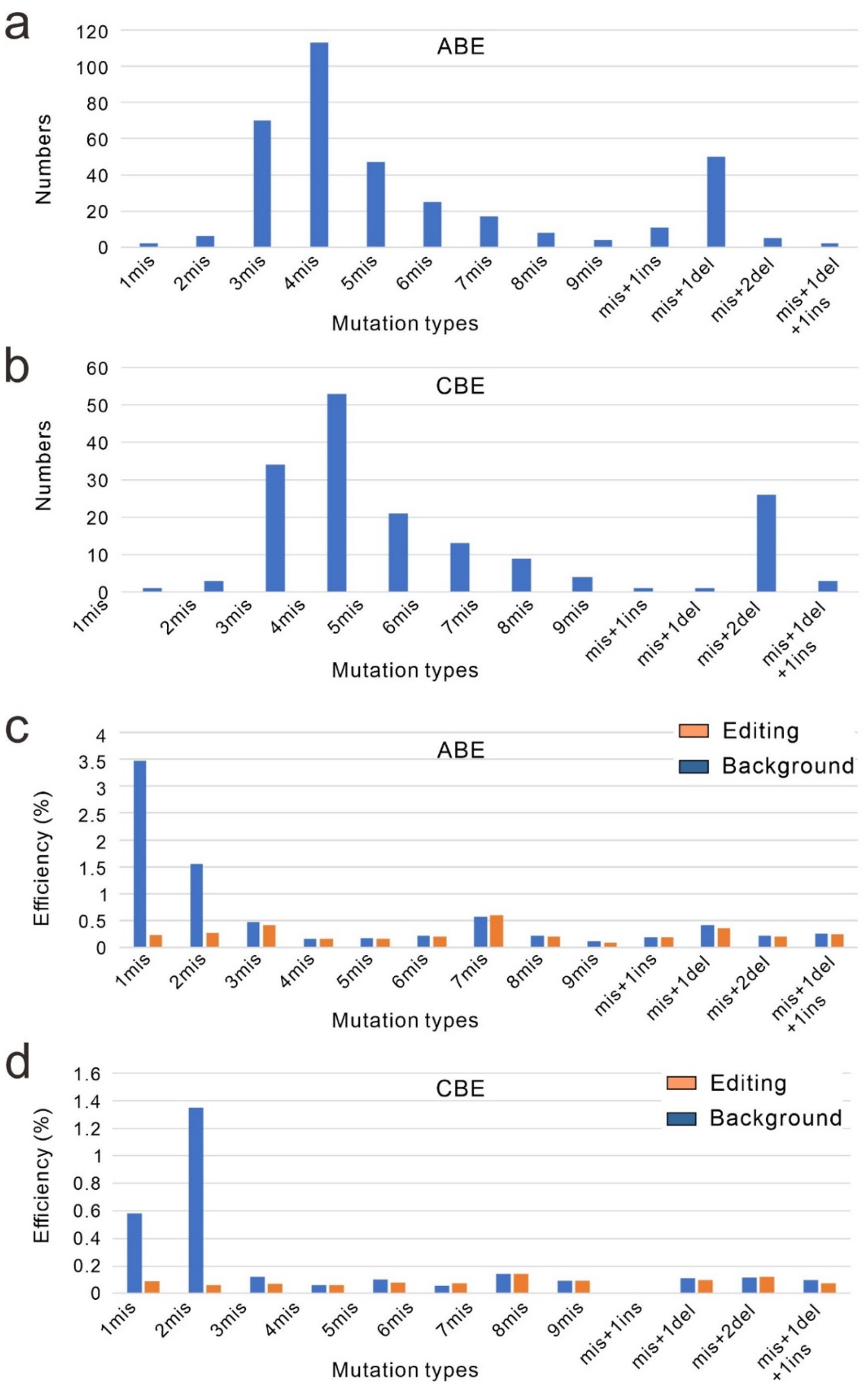

Supplementary Figure 9. Analysis of off-target datasets generated by Kim et al. [1]**.** (a) Mutation types of ABE off-targets generated by Digenome-seq. (b) Mutation types of CBE off-targets generated by Digenome-seq. (c) Average editing efficiency of each mutation type of ABE off-targets. (d) Average editing efficiency of each mutation type of CBE off-targets. Source data are provided as a Source Data file.

Supplementary References

1. Kim D, Kim D-e, Lee G, Cho S-I, Kim J-S. Genome-wide target specificity of CRISPR RNA-guided adenine base editors. *Nature biotechnology* **37**, 430-435 (2019).
