# Supplementary material for: Prediction of base editor off-targets by deep learning
Source: Nat Commun. 2023 Sep 2;14:5358. doi: 10.1038/s41467-023-41004-3 (PMC10475126; doi:10.1038/s41467-023-41004-3)
Supplement: Supplementary file 18 — Description of Additional Supplementary Files [file 41467_2023_41004_MOESM18_ESM.pdf]

Title: Supplementary Data 1.
Description: The efficiency dataset of mutation targets for ABE

Title: Supplementary Data 2.
Description: The efficiency dataset of mutation targets for CBE.

Title: Supplementary Data 3.
Description: The GC content of ABE library.

Title: Supplementary Data 4.
Description: The GC content of CBE library.

Title: Supplementary Data 5.
Description: The independent t-test pvalue (bonferroni correction) across position in ABE.

Title: Supplementary Data 6.
Description: The independent t-test pvalue (bonferroni correction) across position in CBE

Title: Supplementary Data 7.
Description: The benchmark results of various models for ABE

Title: Supplementary Data 8.
Description: The third-party off target datasets of ABE (endogenous)

Title: Supplementary Data 9.
Description: The benchmark results of various models for CBE

Title: Supplementary Data 10.
Description: The third-party off target datasets of CBE (endogenous)

Supplementary Data 11. The third-party off target datasets of ABE (in vitro)

Supplementary Data 12. The third-party off target datasets of CBE (in vitro)

Supplementary Data 13. The sequences of oligonucleotides

Supplementary Data 14. The hyperparameters used for Tree-structured Parzen Estimator
